# Evaluation of Normalization After Implementation of the Digital Dutch Obstetric Telephone Triage System: Mixed Methods Study With a Questionnaire Survey and Focus Group Discussion

**DOI:** 10.2196/33709

**Published:** 2022-06-17

**Authors:** Bernice Engeltjes, Ageeth Rosman, Fedde Scheele, Christiaan Vis, Eveline Wouters

**Affiliations:** 1 Athena Institute for Transdisciplinary Research Faculty of Science VU Amsterdam Amsterdam Netherlands; 2 Department of Healthcare Studies Rotterdam University of Applied Sciences Rotterdam Netherlands; 3 Department of Healthcare Education OLVG Teaching Hospital Amsterdam Netherlands; 4 Department of Neuro & Development Psychology Faculty of Behavioral and Movement Sciences VU Amsterdam Amsterdam Netherlands; 5 Amsterdam Public Health Research Institute – Mental Health Amsterdam Netherlands; 6 Department of Tranzo Tilburg School of Social and Behavioral Sciences Tilburg University Tilburg Netherlands; 7 Department of Allied Health Professions, Health Innovations and Technology Fontys University of Applied Sciences Eindhoven Netherlands

**Keywords:** obstetric triage, Normalization Process Theory, implementation strategy, hierarchy, medical staff

## Abstract

**Background:**

The Dutch Obstetric Telephone Triage System (DOTTS) was developed to improve the quality of acute obstetric care. To achieve optimal effect, the DOTTS should be adopted in the daily care process by triage staff.

**Objective:**

The primary aim was to evaluate the degree of implementation (ie, normalization) of the DOTTS, and the secondary aim was to evaluate which lessons can be learned from its current implementation in Dutch hospitals.

**Methods:**

An evaluation study with a mixed methods design was performed. All triage staff in 9 Dutch hospitals that implemented the DOTTS before September 1, 2019, were invited to complete the Normalization Measure Development (NoMAD) questionnaire between December 2019 and July 2020. The questionnaire is based on the Normalization Process Theory (NPT). This self-reported questionnaire provides insights into the work people do in order to integrate and embed new practice in routine care. The NPT is based on the following 4 constructs: coherence, cognitive participation, collective action, and reflexive monitoring. Within the questionnaire, each construct is represented by 4-7 questions. Questions are scored on a 5-point normalization process scale. Descriptive statistics were used for analysis of questionnaire scores. Subsequently, the questionnaire findings were discussed during a focus group. Template analysis following the 4 constructs was used for analyzing the results of the focus group.

**Results:**

Overall, 173 of 294 (58.8%) triage staff members completed the NoMAD questionnaire, and 90.2% (156/173) of the participants had used the DOTTS for over 6 months. The digital application was used as much as possible or always by 137 of 173 (79.2%) participants. The overall normalization process score was 3.77 (SD 0.36). The constructs coherence, cognitive participation, collective action, and reflexive monitoring scored 4.01 (SD 0.47), 4.05 (SD 0.45), 3.5 (SD 0.45), and 3.72 (SD 0.47), respectively. Analysis of the focus group discussion showed that the added value of the DOTTS was seen as a quality improvement for the care of pregnant women. Dedication of the complete multidisciplinary implementation team was important for facilitating normalization. Support from the medical staff and proper use by all disciplines involved in the triage were seen as facilitating factors. Participants appreciated training and evaluation, and indicated a need for ongoing training and evaluation in relation to goal achievement.

**Conclusions:**

The DOTTS has been integrated into normal care in daily practice. Evaluation by the NoMAD questionnaire provided a positive overall score. These results are in line with or, in some aspects, better than the results of other evaluation studies. Key factors in the normalization process of the DOTTS in obstetric triage are the shared added value for stakeholders, the dedication of the complete multidisciplinary implementation team, and implementation plans that are tailor made in the practical context of the hospital.

## Introduction

The Dutch Obstetric Telephone Triage System (DOTTS) was developed to provide a uniform and practical basis for estimating the severity of symptoms for unplanned obstetric care requests by telephone. In general, a triage system that prioritizes care according to medical urgency has a favorable effect on the safety and efficiency of emergency care [1,2]. The DOTTS is a reliable [3] and valid [4] evidence-based guideline in which presenting symptoms are used to classify the level of urgency from acute hospital admission using transport by ambulance to self-care with advice at home. It was developed through a multiphase multicenter study in consultation with all relevant stakeholders [5]. The stakeholders can be categorized into nursing, medical, and supporting service personnel. In the first category, we included specialized nurses, general nurses, and doctors’ assistants. The second category consisted of obstetricians, obstetricians in training, and midwives. Supporting service personnel consisted of policy makers, managers and management team leaders, and information technology (IT) professionals. All stakeholders were involved in this new activity. The DOTTS has been developed as a digital application and is supported by training of the staff responsible for triage. The DOTTS can be considered as a substantial innovation within the field of obstetric emergency care because it prioritizes care based on the level of urgency in a prestructured manner and not based on the experience of professionals only, it needs the use of digital tools, and it requires changes in the care processes for pregnant women, as well as shifts in roles and responsibilities and improvements in interprofessional collaboration (Multimedia Appendix 1).

Implementation of new innovations in health care should contribute to improve the quality and effectiveness of care [6]. Many innovations are complex and require multiple changes at different levels and by different actors involved in the care processes. When introducing a complex innovation, evaluation of the implementation can optimize this process, and in turn, lessons learned can improve new or further implementation [7,8]. Implementation science has evolved to provide better understanding and explanation of why implementation of innovations succeeds or fails, with the aim to overcome these problems and to improve the methods or the implementation [6]. Numerous theories, models, and taxonomies of implementation have been defined to classify and study implementation [9,10]. To understand the process of implementation, these theoretical approaches can be divided into 3 overarching aims. The first aim is to understand and explain what influences the outcomes of implementation (eg, determinant frameworks, classical theories, and implementation theories). The second aim is to describe and supervise the process of translating research into practice (eg, process models). Finally, the third aim is to evaluate implementation (eg, evaluation frameworks) [11].

In implementation science, attention is paid to the context of implementation [12]. The context can be divided at micro, meso, and macro levels [12-14]. Individual patients and professionals are considered to reflect the micro level. The meso level consists of intraorganizational matters that are characterized by culture and climate, readiness to change, support, and structures within the organization. The macro level is described as the wider environment of exogenous influences, such as policy, guidelines, benchmarking, and the organizational network. Lastly, social relations and support, financial resources, leadership, time availability, evaluation, and physical environment are referred to as being influential at all 3 levels [12-14].

At the organizational level, the Normalization Process Theory (NPT) [15-18] has been developed and added to implementation science. The NPT characterizes implementation as a social process of collective action [15-18]. The NPT offers a framework for process evaluation and for comparative studies of complex interventions. It focuses on factors that promote or inhibit routine embedding of complex interventions in health care practice from a care delivery perspective as opposed to patient- or system-level perspectives. Interactions between the intervention and the way caregivers work in a particular context are seen as core elements of the NPT [15-17]. This theory has been present for some time, has an established scientific basis, and has been evaluated several times for reliability and validity [15-17,19-22]. According to the NPT [15-17], routine use of innovations in practice (ie, the fact that an innovation becomes “normal practice”) can be understood in the following 4 constructs: coherence, cognitive participation, collective action, and reflexive monitoring. The construct coherence considers the clarity of the goal and the importance of the intervention for the individual care provider and among care providers jointly. Cognitive participation describes the way that care providers understand and commit to the working method of the intervention, as well as which work processes have changed as a result of the intervention. The construct collective action considers to what extent sufficient support, training, time, and the actual work to carry out the implementation are experienced. The construct reflexive monitoring describes the extent to which the intervention is evaluated and continues to align with expectations, needs, and progressive understanding (ie, reflection). Together, these 4 constructs provide a heuristic tool for understanding and explaining change processes in health care. In the context of understanding the implementation of the DOTTS within hospitals, we chose to use the NPT as a heuristic tool.

The primary aim of this study was to evaluate the degree of implementation (ie, normalization) of the DOTTS, and the secondary aim was to evaluate which lessons can be learned from its current implementation in Dutch hospitals. This evaluation of the implementation process and the intervention, within the first 9 of 65 (14%) Dutch hospitals, can help to optimize current and new implementations.

## Methods

### Design

An evaluation study of the implementation of the DOTTS in daily practice with a mixed methods design was performed. As methods, a questionnaire survey and a qualitative focus group discussion were used.

### Participating Hospitals and the Context of Implementation

All 9 hospitals that implemented the DOTTS before September 1, 2019, were included in this study. Of the 9 hospitals, 2 were academic hospitals, 5 were teaching hospitals, and 2 were nonteaching hospitals in the Netherlands. Participating hospitals were (1) Erasmus MC Rotterdam, (2) Leiden University Medical Center Leiden, (3) Jeroen Bosch Hospital 's-Hertogenbosch, (4) Antonius Hospital Utrecht, (5) OLVG Amsterdam, (6) Amphia Hospital Breda, (7) Elisabeth Tweesteden Hospital Tilburg, (8) Tjongerschans Hospital Heerenveen, and (9) IJsselland Hospital Capelle aan de IJssel.

In all participating hospitals, the DOTTS was implemented and introduced into routine care. Implementation strategies of the DOTTS were designed for each hospital separately. For this aim, each hospital formed an implementation team with stakeholders. In each hospital, stakeholders involved were at least one nurse and one other care professional (ie, midwife, obstetrician, or obstetrician in training). In most hospitals, implementation teams were much more extensive. The implementation team comprised of a cross-functional team including managers, several nurses, doctors’ assistants from the triage department and outpatient clinic, at least two professionals of the medical team, and an IT professional for adding the digital application of the DOTTS into the electronic patient record system. The implementation team jointly developed a tailored implementation plan, which was an actionable specific work plan for the users of each individual hospital. This work plan included the following steps: researching whether there is support for the innovation, performing a baseline measurement, and formulating relevant goals of triage. In addition to the formulated goals, it was important to organize the right facilities, such as a physical workplace for obstetric triage with a computer, telephone, and headset. In addition, an important step during the implementation process was integrating the digital application of the DOTTS into the hospital’s electronic patient record system. Importantly, in all hospitals, specific training about the DOTTS was given to the staff responsible for triage. In most hospitals, this training was outsourced to an external organization. In some hospitals, this training was given by in-house experts. This choice was determined by the implementation team. Lastly, providing information to third parties before and after implementation was also an important step. The information about the innovation was given to patients, colleagues, and other cooperation partners (eg, general practitioners and the hospital emergency department).

The implementation team went through the implementation strategies before getting started with the DOTTS. The order, as well as the extent of the steps performed, differed per hospital. Progress was evaluated and adjusted through interim process evaluation. The steps were not taken sequentially. Most implementation teams used process steps, which, in retrospect, show similarities with the process models of Kotter or Grol and Wensing [23-25]. These frameworks are intended to support stepwise planning and management of implementation efforts.

### Sample

The participants of this study were users of the DOTTS. Users of the DOTTS were obstetrical nurses, nurses, and doctors’ assistants, and they can be seen as triage staff. Users from all hospitals where the DOTTS was implemented were included. Before inviting participants for the questionnaire survey and the focus group discussion, an exploratory meeting was held with the manager of the department where the participants were employed. In this meeting, the manner of invitation of participants was discussed. Hence, a list of participants was formed. According to the preference of the managers and the intended participants, either email addresses were provided or an information letter including a hyperlink to the questionnaire was forwarded to the participants by the manager. At the end of the questionnaire, the participants were asked if they also wanted to participate in a follow-up study (focus group). After consent, these participants were contacted again for participation in the focus group.

### Measures and Statistical Analysis

#### Questionnaire

The validated [26] Dutch version of the Normalization Measure Development (NoMAD) questionnaire based on the conceptual framework of the NPT was used [20,21,27]. Within the NoMAD questionnaire, each construct of the NPT is represented by 4-7 questions. Questions are answered using the normalization process scale (NPS) as follows: 1, not relevant; 2, strongly disagree; 3, disagree; 4, agree; and 5, strongly agree [26].

In addition to the NoMAD questionnaire, 9 questions for characteristics were added to assess representativeness and distributions. These involved age, professional category (ie, obstetrical nurse, nurse, doctor’s assistant, or other), type of hospital (ie, academic, teaching, or nonteaching), obstetric experience (ie, years), average hours per week spent on triage activities, number of consultations on average per week, start date of the use of the DOTTS, and frequency of the use of the digital application of the DOTTS. All questions were incorporated into an online questionnaire (Qualtrics [28]). Between December 2019 and July 2020, data were collected during a 3-month period in each hospital.

Analyses of participants’ characteristics are presented as numbers or means with percentages or SDs. Descriptive statistics (scale means) were used for the analysis of the questionnaire scores. Analyses of the NPS are presented as numbers with SDs, with minimum and maximum scores. To assess whether the reliability in a different area is sufficient, we also calculated the Cronbach α for the pooled data set. Moreover, the frequency distribution of item responses is presented as the percentage of respondents reporting strongly disagree, disagree, agree, or strongly agree, or respondents who chose to not rate a specific item (not relevant). The questionnaire data analyses were performed in RStudio [29] using psych (scores) [30] and ggplot2 (graphs) [31].

#### Focus Group

Participants of the focus group were triage staff and users of the DOTTS who had completed the questionnaire. A group discussion was held to triangulate and verify the score of the NoMAD questionnaire and the inhibitory and facilitating factors of DOTTS implementation. Participants were asked to discuss whether they recognized and agreed with subscale scores, and needed to come up with possible explanations about differences per construct. Topics were formed based on organizational context factors [12-14] and were structured following the 4 constructs of the NPT [20-22,26]. Context factors were culture and climate, readiness to change, support, policy, guidelines, benchmarking, organizational network, social relations and support, financial resources, leadership, time availability, feedback, and physical environment [12-14].

In April 2021, a digital focus group (Microsoft Teams) meeting was held, recorded, and transcribed verbatim. Atlas-ti [32] was used during template analysis [33] of the focus group results following the 4 constructs of the NPT [20-22,26]. Member check was performed by all participants. Peer-review template analysis [33] was performed with 3 researchers (BE, EMJW, and ANR).

### Ethics Approval

All participants were informed about the study and provided digital informed consent prior to the use of the data for analysis. All data were anonymously processed. Participants were able to withdraw at any time, without any statement of reason. The study was approved by the boards of the Medical Research Ethics Committees United, the Medical Ethics Committee of Leiden University Medical Center, and Erasmus MC of Rotterdam (W.16.053 & P17.075/PG/pg & C1.20191125).

## Results

### Characteristics of the Participants

In total, 294 triage staff members from the 9 hospitals were asked to complete the questionnaire. The overall response rate, after 3 reminders, for complete responses was 58.8% (173/294).

The participants who filled out the questionnaire had a mean age of 43.3 years (SD 11.6 years) and an average work experience in obstetrics of 17.9 years (SD 11.5 years). Participants in the focus group had a mean age of 46 years (SD 9.2 years) and an average work experience in obstetrics of 18.6 years (SD 9.9 years). An overview of the characteristics of the participants is provided in Table 1. In total, 156 of the 173 (90.2%) participants had used the DOTTS for over 6 months. The digital application of the DOTTS was used “as much as possible” or “always” by 137 of the 173 (79.2%) participants.

**Table 1:** Characteristics of the participants.

Characteristic		Questionnaire survey (N=173)	Focus group (N=8)
Age (years), mean (SD)^a^		43.3 (11.6)	46.0 (9.2)
Work experience in obstetrics (years), mean (SD)		17.9 (11.5)	18.6 (9.9)
**Professional category, n (%)^b^**			
	Obstetrical nurse	148 (83.1)	7 (87.5)
	Nurse	6 (3.5)	0 (0)
	Doctor’s assistant	11 (6.4)	1 (12.5)
	Other	8 (4.6)	0 (0)
**Hospital type, n (%)^b^**			
	Academic hospital	67 (38.7)	2 (25.0)
	Teaching hospital	67 (38.7)	4 (50.0)
	Nonteaching hospital	39 (22.5)	2 (25.0)
**Time performing triage (average) per week, n (%)^b^**			
	≥16 hours	49 (36.8)	4 (50.0)
	9-15 hours	50 (31.6)	1 (12.5)
	≤8 hours	74 (31.6)	3 (37.5)
**Number of consultations (average) per week, n (%)^b^**			
	50-100	6 (3.5)	0 (0)
	20-49	20 (11.6)	2 (25.0)
	10-19	55 (31.8)	3 (37.5)
	0-9	90 (52.0)	3 (37.5)
	0	2 (1.2)	0 (0)
**Duration of use of the DOTTS,^c^ n (%)^b^**			
	≥24 months	29 (16.8)	5 (62.5)
	13-24 months	75 (43.3)	3 (37.5)
	6-12 months	52 (30.6)	0 (0)
	≤6 months	17 (9.8)	0 (0)
**Frequency of use of the digital application of the DOTTS, n (%)^b^**			
	Always	48 (27.7)	3 (37.5)
	As much as possible	89 (51.4)	5 (62.5)
	Regularly	21 (12.1)	0 (0)
	Sometimes	13 (7.5)	0 (0)
	Never	2 (1.2)	0 (0)

^a^Missing data (n=1) for age in the questionnaire survey group.

^b^Owing to rounding, the percentages do not add to 100%.

^c^DOTTS: Dutch Obstetric Telephone Triage System.

### Results of the Questionnaire Survey

**Figure 1:**
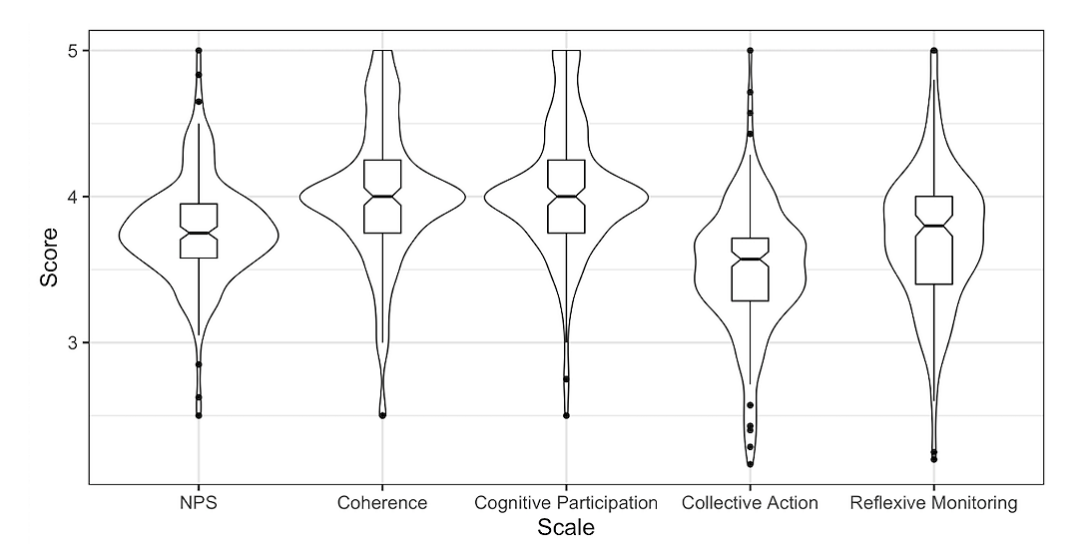
Box plot of the scale scores of the questionnaires. The results are shown as scale scores (2, strongly disagree; 3, disagree; 4, agree; and 5, strongly agree). NPS: normalization process scale.

The overall NPS score was 3.77 (SD 0.36). The constructs coherence, cognitive participation, collective action, and reflexive monitoring scored 4.01 (SD 0.47), 4.05 (SD 0.45), 3.5 (SD 0.45), and 3.72 (SD 0.47), respectively (Table 2). On average, all participants agreed (score 4) with the statements associated with the constructs coherence and cognitive participation. For the constructs collective action and reflexive monitoring, the scores were between 4 (agree) and 3 (disagree). These results were also seen when each hospital was analyzed separately (Multimedia Appendix 2). The scores for the constructs collective action and reflexive monitoring showed more variation compared to the scores for the constructs coherence and cognitive participation (Figure 1). All elements were recognized by most participants. The constructs coherence and cognitive participation had a high percentage of answers with “agree” and “strongly agree” (Multimedia Appendix 3). In the pooled data set, Cronbach α was .85 for the total NPS score and was .71 for coherence, .70 for cognitive participation, .67 for collective action, and .68 for reflexive monitoring (Table 2).

**Table 2:** Overview of Normalization Measure Development (NoMAD) scale scores (N=173).

NoMAD scale	Mean score (SD)	Score range	Cronbach α
Normalization process	3.77 (0.36)	2.5-5.0	.85
Coherence	4.01 (0.47)	2.5-5.0	.71
Cognitive participation	4.05 (0.45)	2.5-5.0	.70
Collective action	3.50 (0.45)	2.2-5.0	.67
Reflexive monitoring	3.72 (0.47)	2.2-5.0	.68

### Results of the Focus Group Discussion

Eight participants (Table 1) discussed the implementation of the DOTTS in their hospitals and what lessons could be learned. The focus group discussion lasted 90 minutes. The focus group was highly valued by the participants and experienced as a reflection moment. Participants discussed whether they agreed with the NPS and construct scores, and came up with possible explanations.

#### Coherence

The added value achieved with the implementation of the DOTTS was considered the improvement of the quality of care services for pregnant women. This corresponds to the construct coherence of the NPT. Participants indicated that implementation of obstetric triage provides uniformity in obstetric emergency care, which underpins a quality improvement for the triage ward. This goal was realized with a dedicated and multidisciplinary implementation team, who, in close cooperation with all users, organized and supervised the implementation of the DOTTS. The multidisciplinary team should consist of representatives from the nursing and medical groups, and commitment and support from the manager are also considered important. The implementation team should be able to create sufficient support and ensure joint ownership of the change. Participants indicated that, among other things, good preparation of the team, sufficient description and clarity of roles and responsibilities, and experience in facilitating implementation were important (Multimedia Appendix 4).

#### Cognitive Participation

To achieve quality improvement, the competencies of triage staff (ie, daily users) should align with the goal of implementation. Dedication, self-efficacy, goal pursuit, and multitasking were mentioned as important competencies to contribute to achieve the added value of the DOTTS. Facilitating factors were clear working agreements for all health care professionals, sufficient capacity of the outpatient clinic organized at the management level, and triage staff who continue to clarify roles and responsibilities of the triage ward with other health care professionals. This corresponds with the construct cognitive participation of the NPT (Multimedia Appendix 4).

#### Collective Action

The added value of the DOTTS was impeded when there was improper use of the obstetric triage ward. Regular outpatient clinic visits, as opposed to real emergencies, were occasionally allowed to be seen at the obstetric triage ward. The reason for perceived improper use of the triage ward by medical staff, referrers, and staff of the outpatient clinic or labor ward, is associated with several factors, including ambiguity in policy between the triage ward and outpatient clinic or labor ward, a lack of capacity in the outpatient clinic, and a decision by medical staff in a hierarchical manner that a regular appointment is to be made at the triage ward. Where the support of medical staff was lacking, this was, in particular, experienced as an important barrier. However, when medical staff were strategically informed and involved by the representative of the implementation group, this barrier was no longer experienced. These reasons correspond to the construct collective action of the NPT (Multimedia Appendix 4).

Organizing adequate training was perceived as supportive of success. Experiences with training varied among the participants, but on every occasion, training contributed to the understanding and implementation of the DOTTS. The participants stated that ongoing training is a facilitating factor in continuous stimulation of daily use. In addition, nurses who work as triage staff need to be well supported in their new task. In addition to performing obstetric triage, appropriate support services, such as administration and equipment, must be facilitated. Group responsibility for such tasks is necessary to foster ongoing ownership and improvement of the service. Developing a sense of responsibility or co-responsibility for the total organization of care and implementation among triage staff is necessary (Multimedia Appendix 4).

#### Reflexive Monitoring

The participants discussed the importance of and the amount of regular evaluation for all stakeholders before, during, and after implementation. Within the different hospitals, there were different experiences with the amount and frequency of evaluation. As users of the DOTTS, participants appreciated evaluation and indicated a need for ongoing evaluation in relation to goal achievement. This corresponds to the construct reflexive monitoring of the NPT (Multimedia Appendix 4).

## Discussion

### Principal Findings

This study aimed to evaluate the use of the DOTTS in daily practice after its implementation in a hospital. Evaluation focused on daily use and on the 4 constructs of the NPS. The DOTTS was used by almost all participants over a period of 6 months or more. The digital application of the DOTTS was used as much as possible or always by most participants. The overall score of the NoMAD questionnaire was 3.77 (SD 0.36). There were some differences per construct, where coherence and cognitive participation scored better and with less variation than collective action and reflexive monitoring. Outcomes of the focus group discussion confirmed the added value of the DOTTS. Use was stimulated by the presence of a dedicated multidisciplinary team and supported by medical staff, as well as proper use of the triage ward, adequate training, and official evaluation.

### Comparison With Prior Work

Our results are in line with and, in some aspects, better than the results of other evaluation studies with complex implementations using the NoMAD questionnaire [26,34,35]. While the Dutch questionnaire was previously applied to e-mental health interventions [26], this is the first time it was applied in obstetrics. To assess whether the reliability in a different area of health care is sufficient, we also looked at the Cronbach α. The results of Cronbach α showed good internal consistency for the total NPS score and acceptable findings for coherence and cognitive participation. However, the results were questionable for the constructs collective action and reflexive monitoring. Our findings are comparable to previous results when using the Dutch version of the questionnaire [26].

Triangulation of our results was facilitated via a focus group discussion. What emerged from this discourse was that triage professionals were able to see the added value (coherence) and were committed (cognitive participation), but struggled with collaboration (collective action) to use the DOTTS and did not always reflect on their efforts (reflexive monitoring) in a systematic manner. A plausible explanation for the favorable results of coherence and cognitive participation is the early and intensive involvement of stakeholders in the development and implementation of the DOTTS, which supports implementation. Stakeholders were involved in the development and gave their commitment about the use of the DOTTS in daily practice. The innovation was created together and is therefore well suited to the needs of care providers [5,36-38]. The construct collective action showed the widest variation. One possible explanation for this variation is the tailor-made approach adopted for the implementation plan. The order, as well as the extent of the steps of the implementation plan, differed per hospital. Moreover, the context differed per hospital, which means that every implementation was also different [12].

The lesson learned from this study is that evaluation (ie, reflection) of preplanned, systematic, and strategic implementation of an innovation in health care deserves more attention. In our study, we mainly evaluated whether the use of the DOTTS normalized after implementation (ie, a state of affairs). Each hospital made a tailor-made plan per implementation, which retrospectively showed similarities with the process models of Kotter or Grol and Wensing [6,23-25]. The change management model by Kotter [25] and the model by Grol and Wensing [6] intended to support the planning and managing implementation efforts. In this study, there was no specific model used; therefore, it is difficult to compare the results with these well-known implementation models. However, evaluation of use, similar to the construct reflexive monitoring, is also an important step in these models.

Improving the quality of services for pregnant women was seen as important after the implementation of the DOTTS. Improvement of quality is mentioned in most implementation science research as a condition for success [14]. Improper use of the triage ward, which was considered by participants as a barrier, is also a well-known phenomenon in the organization of care within the hospital and specifically in triage wards [38-40]. Commonly, in planned hospital care, capacity is limited, causing nonurgent care events to be diverted to the emergency care department. Several factors leading to improper use were also mentioned in the scoping review of Bailey et al [41]. Improper use brings challenges, such as workload stress, which subsequently influence decisions during telephone triage [40,41].

In line with the constructs collective action, cognitive participation, and coherence, implementation by a dedicated multidisciplinary implementation team, which provides guidance during implementation and use of the tool afterwards, was mentioned as important. In our study, this referred to the importance of the involvement of all stakeholders. Preparing an innovation with all stakeholders creates the possibility of optimal support from the start and user friendliness for all stakeholders in daily practice [5,14,42]. Within the multidisciplinary team, special attention should be paid to the participation of the medical group. To change medical staff routines, leadership of the implementation team is an important element [43]. Hierarchy is also a challenging factor here, which is in line with results from other studies indicating that in a hierarchical organization, normalization of an innovation is often more difficult [35]. If the organization of care is arranged by the nurse, it is necessary that it is supported by medical staff [40].

We found that training, which is part of the construct collective action, is an important element of change. This was also seen by existing triage systems [38,44,45]. If users themselves have a need for training because they want to be competent, this contributes to success [46]. After completing the training, it is important to provide continuous evaluation, which contributes to the construct reflexive monitoring, so that the implementation is further optimized and users receive confirmation that they are doing well [38].

### Strengths, Limitations, and Recommendations

The NPT with its validated NoMAD questionnaire was used as an evaluation framework in this study. The NPT is an implementation theory and has been widely used as an evaluation framework [11]. There are also other tools that reflect the success of implementation, such as the Consolidated Framework for Implementation Research (CFIR); Nonadoption Abandonment, Scale-up, Spread, and Sustainability (NASSS) framework; and Reach, Effectiveness, Adoption, Implementation, Maintenance (RE-AIM) framework [11]. The NPT was used in this study because the context of the implementation of an innovation in obstetric care corresponded to the NPT [15].

The use of 2 complimentary research methods is valuable in the interpretation of data. A large group of users were given the opportunity to evaluate the use of the DOTTS, providing a general overview of implementation in several hospitals. The focus group gave the opportunity to triangulate the outcomes, thereby gaining more insight into the meaning of the answers and clarifying the context.

The results are from all hospitals that implemented the DOTTS before September 2019. There was an overall response rate of 58.8% (173/294) from these 9 hospitals. A 50% response rate, which was obtained from all hospitals, can be considered representative [47] (Multimedia Appendix 2). With an average age of 43.3 years and experience of 17.9 years, the sample composition was representative compared to other studies within this profession [48-50]. The participants of the focus group showed good representation of the total research group (Table 1).

To improve the questionnaire, it is recommended to look to the question collective action-2 of the construct collective action because it is the only negatively asked question. It is unclear if every participant interpreted the question correctly.

In this study, we chose to evaluate the degree of normalization after implementation among daily users in 9 hospitals where the DOTTS is offered as usual care. This focus resulted in a lack of information about the theoretical approaches used for each implementation strategy. The tailored implementation strategy created space for context per hospital and the team of stakeholders. However, it limited the ability to evaluate effectiveness per implementation strategy. In addition, due to the current aim of this study, results on the expected quality improvement were lacking. Furthermore, this study only looked at the perspectives of the daily users of the DOTTS. The lack of perspectives of medical staff, outpatient clinic staff, management, and other related professionals is a potential limitation. In view of the importance of tailored implementation strategies, which was highlighted by our research, we recommend that a future study should include representation from the medical group to ensure an inclusive perspective.

Moreover, we did not evaluate the patient perspective. Not every patient will fit into the evidence-based system of the DOTTS, for instance, patients who do not understand self-care with advice. Customization per patient might need to be further developed. The current evaluation was not about these items and requires further research. Further insight into the experiences of patients who have received telephone and physical triage care based on the DOTTS is therefore recommended.

### Conclusions

Normalization of the DOTTS was seen after tailored implementation in 9 hospitals. Key factors in the normalization process of the DOTTS in obstetric triage were (1) the shared added value for stakeholders; (2) the dedication of the complete multidisciplinary implementation team with specific support from medical staff, as well as proper use of the triage ward (as designed) by all disciplines; and (3) implementation plans that are tailor made in the practical context of the hospital. Improvement can be achieved by structuring this process and incorporating implementation strategies, such as systematic training and evaluation, with users.

## Appendix

Multimedia Appendix 1 Video of the Dutch Obstetric Telephone Triage System.

Multimedia Appendix 2 Subanalysis questionnaire findings per hospital.

Multimedia Appendix 3 Frequency distribution of item responses. The constructs are coherence (CO), cognitive participation (CP), collective action (CA), and reflexive monitoring (RM). The upper part of the figure shows the percentage of respondents reporting strongly disagree, disagree, agree, or strongly agree. The gray bar coupled to the y-axis indicates the percentage of participants rating an item as "neutral." The lower part of the figure shows the percentage of respondents who chose to not rate a specific item (not relevant).

Multimedia Appendix 4 Quotes from the focus group discussion. To illustrate the focus group discussion, several quotes per construct from participants are shown. These quotes have been selected by the authors after analysis.

## Abbreviations

DOTTS: Dutch Obstetric Telephone Triage System
IT: information technology
NoMAD: Normalization Measure Development
NPS: normalization process scale
NPT: Normalization Process Theory
