# Supplementary material for: Evaluation of Normalization After Implementation of the Digital Dutch Obstetric Telephone Triage System: Mixed Methods Study With a Questionnaire Survey and Focus Group Discussion
Source: JMIR Form Res. 2022 Jun 17;6(6):e33709. doi: 10.2196/33709 (PMC9250067; doi:10.2196/33709)
Supplement: Multimedia Appendix 2 [file formative_v6i6e33709_app2.docx]

**Multimedia Appendix 2.** Subanalysis questionnaire findings per hospital.

| NoMAD-scale scores  Number of participants  per hospital, n= participants of potentional participants (percentage of total participants per hospitals) | NPS  Score, (SD) | Coherence  Score, (SD) | Cognitive participation  Score, (SD) | Collective Action  Score, (SD) | Reflexive Monitoring Score, (SD) |
| --- | --- | --- | --- | --- | --- |
| Hospital 1 - Academic  n=39 of 79 (49) | 3.68 (0.34) | 3.99 (0.46) | 3.91 (0.47) | 3.42 (0.43) | 3.63 (0.43) |
| Hospital 2 - Academic  n=28 of 51 (55) | 3.92 (0.4) | 4.08 (0.51) | 4.14 (0.45) | 3.73 (0.48) | 3.86 (0.55) |
| Hospital 3 - Teaching  n= 25 of 38 (66) | 3.75 (0.38) | 4.01 (0.45) | 4.01 (0.5) | 3.53 (0.35) | 3.64 (0.52) |
| Hospital 4 - Teaching  n= 14 of 16 (88) | 3.72 (0.32) | 4.16 (0.43) | 4.15 (0.29) | 3.21 (0.52) | 3.74 (0.43) |
| Hospital 5 - Teaching  n= 12of 14 (86) | 3.66 (0.26) | 3.71 (0.56) | 3.92 (0.36) | 3.56 (0.18) | 3.55 (0.42) |
| Hospital 6 - Teaching  n= 12 of 17 (59) | 3.71 (0.21) | 3.85 (0.29) | 4.12 (0.34) | 3.36 (0.62) | 3.75 (0.26) |
| Hospital 7 - Teaching  n= 6 of 10 (60) | 3.38 (0.56) | 3.54 (0.64) | 3.83 (0.68) | 2.9 (0.51) | 3.53 (0.71) |
| Hospital 8 - Non-Teaching n= 20 of 29 (69) | 3.82 (0.29) | 4.05 (0.32) | 4.18 (0.34) | 3.52 (0.32) | 3.77 (0.45) |
| Hospital 9 - Non-Teaching  n=19 of 40 (48) | 3.96 (0.3) | 4.25 (0.4) | 4.2 (0.48) | 3.68 (0.33) | 3.92 (0.35) |
| Pooled total  n=173 of 294 (59) | 3.77 (0.36) | 4.01 (0.47) | 4.05 (0.45) | 3.5 (0.45) | 3.72 (0.47) |

Total score of Normalization Process Theory expressed by Normalization Process Score (NPS). Score per construct of Normalization Process Theory: Coherence, Cognitive Participation, Collective Action and Reflexive Monitoring. Scale scores represented in Mean and standard deviation (SD), 2: strongly disagree, 3: disagree, 4: agree and 5: strongly agree. Participating hospitals were: 1. Erasmus MC Rotterdam, 2. Leiden University Medical Center Leiden, 3. Jeroen Bosch hospital ’s Hertogenbosch, 4. Antonius hospital Utrecht, 5. OLVG Amsterdam, 6. Amphia Hospital Breda, 7. Elisabeth Tweesteden hospital Tilburg, 8. Tjongerschans hospital Heerenveen, 9. IJsselland hospital Capelle aan de IJssel.
