# Supplementary figures and images for: Evaluation of Normalization After Implementation of the Digital Dutch Obstetric Telephone Triage System: Mixed Methods Study With a Questionnaire Survey and Focus Group Discussion
Source: JMIR Form Res. 2022 Jun 17;6(6):e33709. doi: 10.2196/33709 (PMC9250067; doi:10.2196/33709)

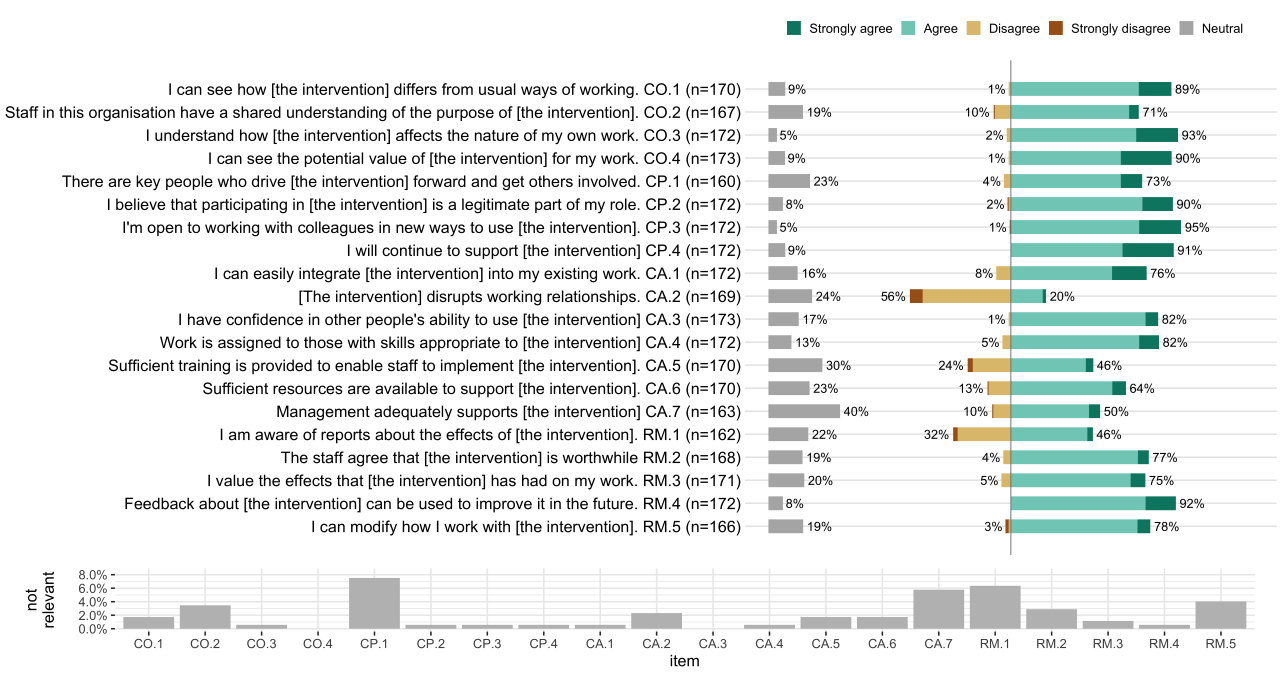

Supplement: Multimedia Appendix 3 [file formative_v6i6e33709_app3.png]
