# Supplementary material for: Evaluation of Normalization After Implementation of the Digital Dutch Obstetric Telephone Triage System: Mixed Methods Study With a Questionnaire Survey and Focus Group Discussion
Source: JMIR Form Res. 2022 Jun 17;6(6):e33709. doi: 10.2196/33709 (PMC9250067; doi:10.2196/33709)
Supplement: Multimedia Appendix 4 [file formative_v6i6e33709_app4.docx]

**Multimedia Appendix 4.** Quotes from focus group discussion.

| **Coherence**  Colleagues already received these calls, only now everyone takes the calls in the same way and treats people in the same way. ..... Well, it is of course true that people have to take the step, you really have to sit behind that computer to fill in the digital triage application directly while you are making a phone call. (participant 2)  In the end, it is documented; that's also a plus. (participant 5)  **Cognitive participation**  …… From scratch, and that we all put our shoulders under the [ new ] triage together. Therefore we are a very well-functioning triage department. As a result, everyone becomes more and more enthusiastic and remains more involved, because we have built that together. I think this is really the strength with us. (participant 7)  It is a change that has been handled very well, while that is still a point of attention for us. That you work out and implement something and then it fade again, and everyone starts doing their own thing again. But there really is a "before" and "after" here. (participant 1) **Collective Action** As nurses, we can have a specific opinion and add structure to it, but then we heavily run into [ routine of ] obstetricians. We can say [ to them ] that there is a maximum of so many [ patients ] on our acute care department. But then again, entire outpatients’ clinics are simply scheduled with us at the acute care department. (participant 2)  And we haven't had any training either. Very unfortunate of course. We have done some sort of training on-the-job, so a small group has been trained, including X and myself. And we will then just work our services and while working you explain that we are now going to work with DOTTS. That went fine in itself, but I'm missing a piece, well, continuity. And such a refresher course after 2 years would also be very good for us. So that there is a little more structure in it. (participant 2) **Reflexive monitoring** We also evaluated very often in the beginning; when it was started. And now the last year (1 ½ years) nothing more. (participant 3)  Well with us, if you run into problems, you can submit it at a general team meeting the [ no specific evaluation]. (participant 6)  …..there is no evaluation. We work with it, and then it's done. (participant 2) |
| --- |
